# Pan-Cancer Analysis Identifies Liver Metastases as Negative Predictive Factor for Immune Checkpoint Inhibitors Treatment Outcome

**DOI:** 10.3389/fimmu.2021.651086

**Published:** 2021-06-24

**Authors:** Xiao-Juan Chen, Aiqun Ren, Liang Zheng, En-Dian Zheng, Tao Jiang

**Affiliations:** ^1^ Department of Gastroenterology, Wenzhou People’s Hospital, Wenzhou Third Clinical Institute Affiliated to Wenzhou Medical University, Wenzhou, China; ^2^ Department of Pulmonary Medicine, Shanghai Respiratory Research Institute, Zhongshan Hospital, Fudan University, Shanghai, China; ^3^ Department of Medical Oncology, Shanghai Pulmonary Hospital & Thoracic Cancer Institute, Tongji University School of Medicine, Shanghai, China

**Keywords:** pan-cancer, liver metastases, immune checkpoint inhibitor, prognosis, treatment outcome

## Abstract

This study aimed to investigate the predictive value of liver metastases (LM) in patients with various advanced cancers received immune-checkpoint inhibitors (ICIs). First, clinical and survival data from a published cohort of 1,661 patients who received ICIs therapy were downloaded and analyzed. Second, a retrospective review of 182 patients with advanced non-small-cell lung cancer (NSCLC) who received PD-1/PD-L1 monotherapy was identified. Third, a meta-analysis of published trials was performed to explore the impact of LM on the efficacy of anti-PD-1/PD-L1 based therapy in advanced lung cancers. Pan-cancer analysis revealed that patients with LM had significantly shorter overall survival (OS) than those without LM (10 *vs.* 20 months; *P < *0.0001). Subgroup analysis showed that the presence of LM was associated with markedly shorter OS than those without LM in ICI monotherapy group (*P < *0.0001), but it did not reach the statistical significance in ICI-based combination therapy (*P* = 0.0815). In NSCLC, the presence of LM was associated with significantly inferior treatment outcomes in both pan-cancer and real-world cohort. Interestingly, ICI-based monotherapy and combination therapy could simultaneously prolong progression-free survival (PFS) and OS than chemotherapy in patients without LM. However, ICI-based monotherapy could not prolong PFS than chemotherapy in patients with LM while ICI-based combination therapy could dramatically prolong both PFS and OS. Together, these findings suggested that the presence of LM was the negative predictive factor in cancer patients received ICIs monotherapy, especially in NSCLC. ICI-based combination therapy might overcome the intrinsic resistance of LM to ICIs while the optimal combinatorial strategies remain under further investigation.

## Introduction

The liver is a large and very vascular glandular organ of human beings, which secretes bile and causes important biological changes in many of the substances contained in the blood (1, 2). It is also the main sites of distant metastases in patients with advanced cancers including melanoma, gastrointestinal cancer, breast cancer, as well as lung cancer (3, 4). Approximately 15–40% of patients with advanced cancers would be diagnosed with liver metastases (LM) during his/her lifetime (5, 6). Patients with LM often have an unsatisfactory prognosis (7). To make matters worse, several previous publications revealed that the presence of LM was a negative predictive factor for molecular targeted therapy in patients with driver gene mutations (e.g. EGFR) (8), indicating that alternative treatment strategy is warranted.

Immune-checkpoint inhibitors (ICIs) targeting cytotoxic T lymphocyte-associated antigen 4 (CTLA-4), programmed cell death 1 (PD-1) and its ligand (PD-L1) interaction have shifted the treatment landscape of advanced cancers and significantly improved the overall survival (OS) (9–12). Currently, ICI is one of the key and standard treatment strategies for various solid tumors. Nevertheless, several recent studies reported that patients with LM cannot benefit from ICI monotherapy (13, 14). Osorio et al. analyzed 761 individual lesions from 214 patients with non-small-cell lung cancer (NSCLC) and 290 lesions from 78 patients mismatch repair deficiency (MMRD) carcinoma treated with PD-1 monotherapy and found that LM had the least responses (15). However, other studies reported that LM did not compromise the survival benefit of patients received ICIs (16, 17). These contrary findings indicated that the predictive value of LM for ICIs treatment remains further investigation.

Therefore, we performed this pan-cancer analysis to investigate the predictive value of LM in patients with various advanced cancers received ICIs. We also analyzed a real-world cohort and conducted a systematic review with meta-analysis to explore the impact of LM on the efficacy of anti-PD-1/PD-L1 based treatment in advanced lung cancers.

## Methods

### Data Identification and Pan-Cancer Analysis

To investigate the impact of LM on ICIs treatment outcome, we downloaded the pan-cancer clinical and survival data from a recently published cohort of 1,661 patients treated with ICIs therapy from the cBioPortal online database (https://www.cbioportal.org) (18–20). Firstly, we analyzed the predictive significance of LM in all included patients with various cancers. Then, we explored the predictive value of LM for ICIs treatment outcomes in several common types of solid tumors including melanoma, colorectal cancer and NSCLC. We also compared the tumor mutational burden (TMB) level between patients with and without LM. Similar to previous study, TMB was defined as the total number of nonsynonymous mutations including somatic, coding, base substitution, and indel mutations per megabase (mut/Mb) of genome examined.

### Patients’ Selection in a Real-World Cohort

To further assess the impact of LM for ICI treatment outcome in NSCLC, we performed a retrospective review of the patients diagnosed with advanced NSCLC who received anti-PD-1/PD-L1 monotherapy from January 1, 2016 to November 1, 2020 in two medical centers. The major inclusion criteria were (i) histological or pathological confirmation of advanced NSCLC, (ii) radiological confirmation of LM including magnetic resonance imaging (MRI) and/or enhanced computed tomography (CT), and (iii) evaluable for treatment response assessment. Firstly, patients with initial diagnosis of stage IV NSCLC were identified. Then, patients with LM and sufficient clinical information were selected. Other distant metastases were detected by using whole body positron emission tomography (PET) or PET/CT, cranial and thoracic CT/MRI, abdominal ultrasound or bone scan. All of them had received anti-PD-1/PD-L1 antibodies as monotherapy, regardless of treatment lines. The dose of each type of anti-PD-1/PD-L1 antibodies was used according to the recommended dose from drug instructions or phase II/III trials. This study was conducted in accordance with the provisions of the Declaration of Helsinki and was approved by the ethics committee of each medical center.

### Data Collection

The major clinicopathological parameters including age, sex, smoking history, Eastern Cooperative Oncology Group performance status (ECOG PS), lung cancer histology (WHO classification) (21), sites of metastasis, therapeutic regimens and treatment lines were collected. Smoking status, ECOG PS and age were recorded at the time of initial diagnosis. A never smoker was defined as a person who had smoked less than 100 cigarettes during his/her lifetime. Which anti-PD-1/PD-L1 antibodies were selected according to clinical treatment guidelines or by the investigators’ or patients’ discretion. Response including complete response (CR), partial response (PR), stable disease (SD) and disease progression (PD) was assessed using Response Evaluation Criteria in Solid Tumors version 1.1. Progression-free survival (PFS) was assessed from the date the patient began ICI treatment to the date of PD or death of any cause. Patients who had not progressed were censored at the date of their last follow-up. OS was calculated from the beginning of immunotherapy to the date of death of any cause. Patients who were still alive or lost contact were censored at the date of last scan. The last follow-up was December 1, 2020.

### Meta-Analysis of Published Trials

We performed a publication search of the PubMed/Medline, EMBASE, Google Scholar, Cochrane Library, and Web of Science databases through December 31, 2020, using “lung cancer” and “PD-L1” and “liver metastasis” and their related words. Data on the relationship between liver metastasis and OS or PFS in NSCLC patients treated with anti-PD-1/PD-L1 based treatments were collected from publications meeting the eligibility criteria. The details of our methodology are described in the **Supplemental Material**.

### Statistical Analysis

Clinicopathologic characteristics were descriptively summarized by number and percentages. The categorical variables were compared by using chi-square test, or Fisher’s exact test when needed. The continuous variables were analyzed by ANOVA and/or Tukey’s multiple comparison tests. The difference of baseline features between different treatment groups was compared with the χ^2^ test. PFS was defined as the time from the date of initiation of ICIs based treatment to the date of systemic progression or death and was censored at the date of last tumor assessment (when carried out). OS was calculated from the date of ICIs based treatment start to the date of death of any cause or last follow-up. Kaplan–Meier curves with two-sided log-rank tests and Cox proportional hazards model with calculated hazard ratios (HRs) and 95% confidence intervals (CIs) were used to determine the survival difference. All *P* values were two-sided and considered significant at *P < *0.05. All statistical analyses were performed using the SPSS statistical software, version 20.0 (SPSS Inc., Chicago, IL, USA).

## Results

### Pan-Cancer Analysis

We identified a cohort of 1,661 cancer patients with 11 cancer types. Among them, 139 (8.4%) cases had LM. Baseline features of included patients were listed in **Table 1**. Totally, 1,034 (62.3%) male patients were included, and 739 (44.5%) cases had age ≥65 years. Most of them received PD-1/PD-L1 inhibitors treatment (78.7%). There was a significantly higher rate of patients received ICI-based combination therapy in patients with LM than those without LM (*P* = 0.018).

**Table 1:** Baseline characteristics of the study population.

Variables	All	Liver metastasis	No. liver metastasis	*P* value
**Total**	1,661	139	1,522	
**Age at diagnosis**				
<65 years	922	80	842	0.612
≥65 years	739	59	680	
**Gender**				
Male	1,034	83	951	0.519
Female	627	56	571	
**Cancer type**				
Bladder Cancer	215	13	202	—
Breast Cancer	44	6	38	
Cancer of Unknown Primary	88	13	75	
Colorectal Cancer	110	26	84	
Esophagogastric Cancer	126	9	117	
Glioma	117	0	117	
Head and Neck Cancer	139	8	131	
Melanoma	320	31	289	
Non-Small Cell Lung Cancer	350	31	319	
Renal Cell Carcinoma	151	2	149	
Skin Cancer, Non-Melanoma	1	0	1	
**Drug type**				
Combination	255	31	224	0.018
CTLA-4 inhibitor	99	10	89	
PD-1/PD-L1 inhibitor	1,307	98	1,209	

CTLA-4, cytotoxic T-lymphocyte-associated protein 4; PD-1, programmed cell death protein 1; PD-L1, programmed cell death protein ligand 1.

**Figure 1:**
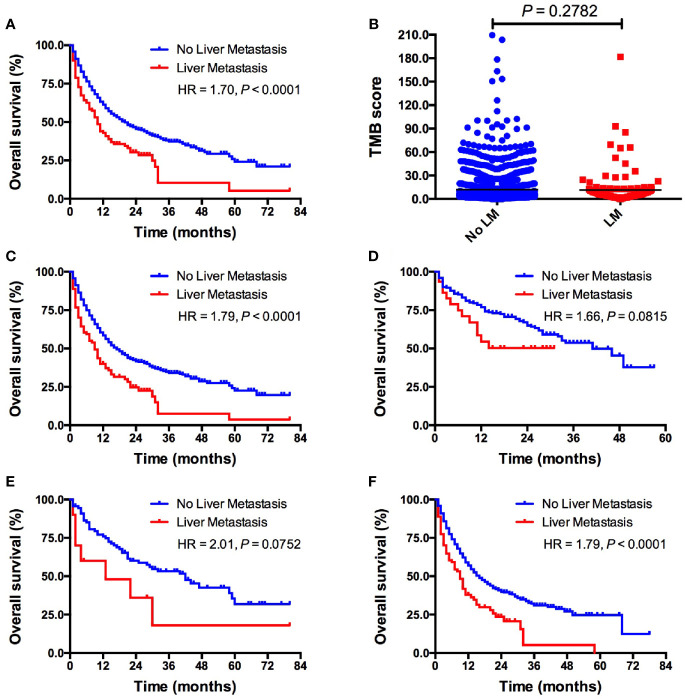
Pan-cancer analysis of the predictive value of LM for ICIs treatment outcomes. **(A)** OS comparison between patients with *vs.* without LM in whole cohort; **(B)** TMB level comparison between patients with *vs.* without LM in whole cohort; **(C)** OS comparison between patients with *vs.* without LM in ICIs monotherapy group; **(D)** OS comparison between patients with *vs.* without LM in ICIs based combination therapy group; **(E)** OS comparison between patients with *vs.* without LM in PD-1/PD-L1 monotherapy group; **(F)** OS comparison between patients with *vs.* without LM inCTLA-4 monotherapy group. LM, liver metastasis; TMB, tumor mutational burden; ICI, immune checkpoint inhibitor.

Patients with LM had significantly shorter OS than those without LM (10 *vs.* 20 months; HR = 1.70, *P < *0.0001; **Figure 1A**) in all included patients. Intriguingly, TMB level was comparable between patients with and without LM (5.6 *vs.* 6.1, *P* = 0.2782; **Figure 1B**). Subgroup analysis showed that patients with LM also had markedly inferior OS than those without LM (9 *vs.* 17 months; HR = 1.79, *P < *0.0001; **Figure 1C**) in ICI monotherapy group. However, the presence of LM was associated with inferior OS in ICI combination therapy without statistical significance (not reached *vs.* 41 months; HR = 1.66, *P* = 0.0815; **Figure 1D**). Interestingly, in patients treated with PD-1/PD-L1 monotherapy, the presence of LM was associated with significantly shorter OS (9 *vs.* 16 months; HR = 1.79, *P < *0.0001; **Figure 1F**). Whereas the presence of LM was associated with inferior OS in CTLA-4 monotherapy but it did not reach the statistical significance (13 *vs.* 42 months; HR = 2.01, *P* = 0.0752; **Figure 1E**) mainly due to small sample size. We also investigated the predictive value of LM in several specific types of tumors. The presence of LM was associated with obviously worse OS in colorectal cancer (*P* = 0.0289; **Supplemental Figure S1A**) and NSCLC (*P* = 0.0449; **Supplemental Figure S1C**) group than those without LM, but it did reach the statistical significance in melanoma cohort (*P* = 0.0668; **Supplemental Figure S1B**). Multivariate analysis revealed that LM was significantly associated with worse OS (*P <* 0.001; **Table 2**). Additionally, ICIs based combination therapy and high tumor purity was significantly associated with longer OS (*P <* 0.001, *P* = 0.042, respectively; **Table 2**).

**Table 2:** Multivariate analyses of clinical parameters on OS.

Factor	HR (log rank)	95% CI	*P* value
Age (< 65/≥ 65)	1.003	0.873–1.152	0.971
Sex (Female/male)	1.116	0.971–1.284	0.122
Drug (monotherapy/combination)	1.797	1.450–2.227	<0.001
Liver metastasis (yes/no)	1.666	1.335–2.078	<0.001
Muation count (<median/>median)	1.338	1.072–1.669	0.01
TMB score (<median/>median)	1.050	0.844–1.305	0.662
Tumor purity (<50/>50)	1.153	1.005–1.332	0.042

HR, hazard ratio; CI, confidence interval; TMB, tumor mutational burden.

### Baseline Features of Included Patients in Real-World Cohort

To further assess the predictive value of LM in patients with advanced NSCLC, we identified a total of 182 NSCLC patients received PD-1/PD-L1 monotherapy from January 1, 2016 to November 1, 2020 in two medical centers. Around 23 (18.0%) of them were initially diagnosed with LM. The clinical characteristics of the study population were summarized in **Table 3**. In total, 146 (80.2%) male patients were included, and the mean age was 61 years. Most of them were smokers (58.8%) and had performance status of ECOG 1-2 (91.2%). Adenocarcinoma is the most common histological type (58.8%). Some 53 (29.1%) patients received PD-1/PD-L1 monotherapy as first-line therapy.

**Table 3:** Baseline characteristics of the population from real-world cohort.

Variables	All	Liver metastasis	No liver metastasis	*P* value
**Total**	182	23	159	
**Age at diagnosis**				
< 65 years	109	13	96	0.724
≥ 65 years	73	10	63	
**Gender**				
Male	146	17	129	0.417
Female	36	6	30	
**Smoking history**				
Never	75	11	64	0.490
Ever/current	107	12	95	
**ECOG PS**				
0	16	3	13	0.707
1–2	166	20	146	
**Stage**				
IIIB	12	2	10	0.988
IV	170	21	149	
**Histological type**				
Adenocarcinoma	107	10	97	0.110
Squamous cell carcinoma	51	8	43	
Others	24	5	19	
**Treatment line**				
First	53	4	49	0.281
Second or above	129	19	110	

ECOG PS, Eastern Cooperative Oncology Group performance status.

### The Predictive Value of LM in Real-World Cohort

**Figure 2:**
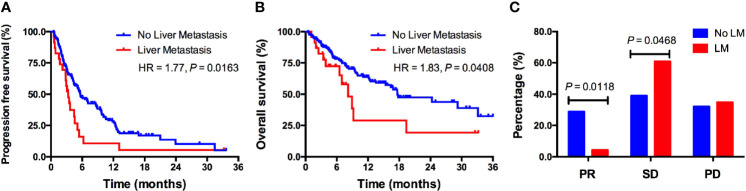
The predictive value of LM for ICIs treatment outcomes in a real-world cohort. **(A)** Kaplan–Meier curve of PFS in patients with versus without LM; **(B)** Kaplan–Meier curve of OS in patients with versus without LM; **(C)** Response rate comparison between patients with versus without LM. LM, liver metastasis; PR, partial response; SD, stable disease; PD, disease progression.

Survival analyses using the Kaplan–Meier method and log-rank test showed significantly shorter PFS in patients with LM received PD-1/PD-L1 monotherapy compared to patients without LM (3.3 *vs.* 5.6 months; HR = 1.77, *P* = 0.0119; **Figure 2A**). Patients with LM also had significantly shorter OS than those without LM (8.2 *vs.* 17.6 months; HR = 1.83, *P* = 0.0408; **Figure 2B**). The objective response rate (ORR) was significantly lower in patients with LM than in patients without LM (4.3% *vs.* 28.9%, *P* = 0.0118; **Figure 2C**). The disease control rate (DCR) was similar between two groups (65.2% *vs.* 67.9%; **Figure 2C**). In multivariate analysis, LM was significantly associated with both shorter PFS (HR = 1.546, *P* = 0.039; **Supplemental Table S2**) and OS (HR = 1.543, *P* = 0.046; **Supplemental Table S1**). Additionally, PD-1/PD-L1 monotherapy as first-line treatment was significantly associated with longer PFS (*P* = 0.020; **Supplemental Table S1**) and OS (*P* = 0.027; **Supplemental Table S1**).

### Features of Included Publication in the Meta-Analysis

Considering the negative predictive value of LM in NSCLC from both the online database and real-world cohort, we conducted a meta-analysis to compare the different treatment outcomes of anti-PD-1/PD-L1 based therapies in NSCLC with versus without LM. As shown in **Supplemental Figure S2**, 298 potentially relevant studies were screened. Most of the excluded publications were reviews, comments, duplications, or studies with incomplete data. The current study assessed 6,274 cases from 11 publications to investigate the distinct treatment outcomes of anti-PD-1/PD-L1 based therapies in NSCLC with versus without LM (22–32). The main features of the eligible studies are shown in **Supplemental Table S2**. Each included trial had the excellent methodologic quality (**Supplemental Table S3**).

### Treatment Outcomes in NSCLC With Versus Without LM

**Figure 3:**
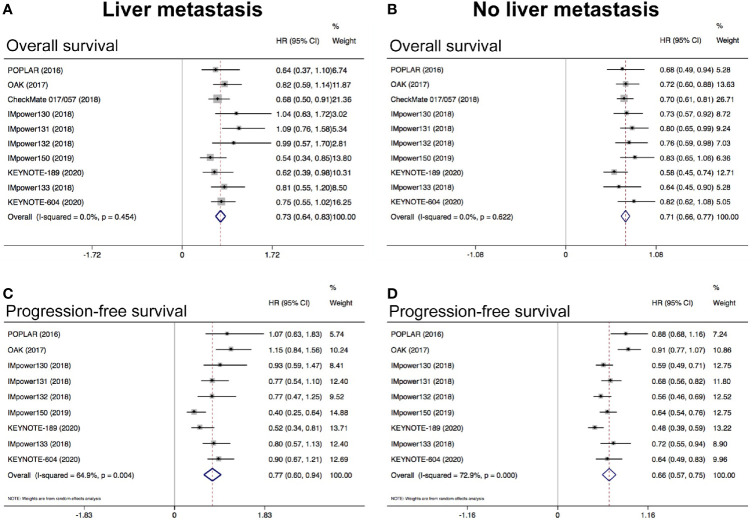
Meta-analysis to evaluate the predictive value of LM in NSCLC treated with ICIs. **(A)** Pooled analysis of OS in patients with LM; **(B)** Pooled analysis of OS in patients without LM; **(C)** Pooled analysis of PFS in patients with LM; **(D)** Pooled analysis of PFS in patients without LM. LM, liver metastasis.

The pooled results showed that anti-PD-1/PD-L1 based therapies was correlated with better OS (HR = 0.73, 95% CI: 0.64–0.83; *P <* 0.05; **Figure 3A**) and PFS (HR = 0.77, 95% CI: 0.60–0.94; *P <* 0.05; **Figure 3C**) when compared with standard chemotherapy in patients with LM. Similarly, the pooled results indicated that anti-PD-1/PD-L1 based therapies was associated with longer OS (HR = 0.71, 95% CI: 0.66–0.77; *P <* 0.05; **Figure 3B**) and PFS (HR = 0.66, 95% CI: 0.57–0.75; *P <* 0.05; **Figure 3D**) in patients without LM. Both results of OS showed low heterogeneity (I^2^ = 0.0%, *P* = 0.454; I^2^ = 0.0%; *P* = 0.622; respectively), but results of PFS showed high heterogeneity (I^2^ = 64.9%, *P* = 0.004; I^2^ = 72.9%; *P <* 0.001; respectively). Subgroup analysis revealed that anti-PD-1/PD-L1 monotherapy could not prolong PFS than chemotherapy in patients with LM while anti-PD-1/PD-L1 based combination therapy could significantly prolong PFS (**Supplemental Figure S3**). In patients without LM, both anti-PD-1/PD-L1 based monotherapy and combination therapy could simultaneously prolong PFS and OS (**Supplemental Figure S3**).

## Discussion

The present study reported that the presence of LM was correlated with significantly inferior treatment outcomes in ICI based monotherapy. However, it was not associated with significantly inferior OS in ICI based combination treatment group. In one of the most common solid tumors, the presence of LM was associated with significantly inferior treatment outcomes in patients with advanced NSCLC from both the pan-cancer and real-world cohort. Interestingly, meta-analysis revealed that anti-PD-1/PD-L1 based monotherapy and combination therapy could simultaneously prolong PFS and OS in NSCLC patients without LM. However, anti-PD-1/PD-L1 based monotherapy could not prolong PFS than chemotherapy in NSCLC patients with LM while anti-PD-1/PD-L1 based combination therapy could dramatically prolong both PFS and OS. Collectively, these findings indicate that the presence of LM was the negative predictive factor in patients with advanced cancers received ICIs monotherapy. ICI based combination therapy might overcome the intrinsic resistance of LM to ICI monotherapy while the optimal combinatorial strategies need further investigation.

As one of the most common distant metastasis in solid tumors, LM has unique the tumor immune microenvironment (3, 4). When LM-competent cells entered the liver, they would encounter a variety of cells including liver sinusoidal endothelial cells, liver-associated lymphocytes, Kupffer cells, hepatic stellate cells, dendritic cells, and portal fibroblasts (3, 4). All of them would have an impact on the biology of LM formation and progression. Previously, several elegant studies have unraveled that liver could promote the specific immune tolerance under the circumstance of viral infections, organ transplantation and autoimmune diseases *via* eliminating effector T cell, inducing effector T cell anergy and regulatory T cells (Tregs) (33–35). Whether LM could impair the systemic antitumoral immunity and ICI treatment outcomes remains unknown. Recently, several publications investigated the predictive value of LM for ICI efficacy. Paul et al. analyzed 336 patients with melanoma or NSCLC received pembrolizumab and reported that LM was associated with significantly reduced responses and PFS (13). Subsequently, a series of studies reported the negative predictive value of LM for ICI treatment in specific types of solid tumors (16, 36). Furthermore, our study indicated that the presence of LM was the pan-cancer negative predictive factor in patients received ICIs monotherapy. Interestingly, our data revealed that ICI based combination therapy could dramatically prolong both PFS and OS in patients with LM and the presence of LM did not significantly impair the efficacy of ICI based combination therapy. Taken together, these findings suggested that ICI monotherapy is insufficient to control the disease in patients with cancer and LM. Reasonable ICI based combination therapy need future investigation in this clinical scenario.

To unravel the mechanism of liver antitumoral immune tolerance in the context of cancer is the key to improve the clinical practice and prognosis of patients with LM. Several recent publications shed a light on this research area. Zhou et al. reported that LAG3 blockade could increase proliferation and effector cytokine production of intratumoral T-cells isolated from LM of colorectal cancer in response to both polyclonal and autologous tumor-specific stimulations, suggesting a new promising immunotherapeutic target for LM of colorectal cancer (37). James et al. observed that the presence of liver could suppress the systemic antitumor immunity in a dual-tumor immunocompetent mouse model (38). Mechanistically, coordinated activation of Tregs and modulation of intratumoral CD11b+ monocytes led to the antigen specific immune suppression. While Tregs were depleted or destabilized by using specific inhibitors, the antitumoral immune of PD-1 antibody could resuscitate within LM. More recently, Yu et al. found that LM could siphon activated CD8+ T cells from systemic circulation and induce antigen-specific Fas+CD8+ T cells undergo apoptosis following their interaction with FasL+CD11b+F4/80+ monocyte-derived macrophages (39). These immunosuppressive hepatic macrophages could be eliminated by liver-directed radiotherapy, which result in the increase of hepatic T cell survival and decrease of hepatic siphoning of T cells. These two elegant study together suggested the specific immune microenvironment of LM and ICI based combination therapy (e.g. plus CTLA-4 inhibitor, EZH2 inhibitors, radiotherapy, etc.) could rescue systemic antitumor immunity and improve the prognosis of cancer patients with LM.

These current findings had several significant limitations that should be acknowledged and treated with caution. First, relatively small number of eligible patients into the final real-world cohort analysis and the retrospective nature will inevitably have several biases such as selection bias. Meta-analysis is the archetypical observation and heterogeneous clinical trials were included without any technically correct information, making it not necessarily meaningful. Thus, the present findings must be cautiously interpreted and large-scale prospective study is eagerly warranted. Second, since PD-L1 expression results from online database was unavailable and real-world cohort did not record the PD-L1 expression, the impact of PD-L1 expression on the treatment outcomes could not be investigated. Third, details of patients with LM in published trials were not reported, making further subgroup analysis difficult. Last but not least, the mechanisms of LM conferring poor prognosis in patients treated with ICI are not well stated. Since it is much difficult to obtain the paired primary and liver metastatic lesions in clinical practice, we cannot include any specific exome and/or transcriptomic features in the multivariate analysis. Therefore, currently, we cannot make a solid conclusion on the true predictive or prognostic significance of LM. In the future, we need comprehensively study the multi-omic features including genomic, transcriptomic, proteomic, metabolic and epigenomic features, especially single-cell transcriptome analysis and TCR sequencing of both primary lesions and LM to unravel the impact of specific immune clusters (e.g. macrophages, CD8+ T cells, Tregs, etc.) on tumor progression in the liver and ICI response, and then establish the true predictive or prognostic significance of LM in patients received ICIs therapy.

In conclusion, the current study indicated that the presence of LM was the negative predictive factor in cancer patients received ICIs monotherapy. ICI based combination therapy could dramatically prolong both PFS and OS in patients with LM and the presence of LM did not significantly impair the efficacy of ICI based combination therapy, suggesting it might overcome the intrinsic resistance of LM to ICIs monotherapy. However, due to the limited clinical and survival data from this study, the optimal combinatorial strategies in patients with LM are still unknown.

## Data Availability Statement

The raw data supporting the conclusions of this article will be made available by the authors, without undue reservation.

## Ethics Statement

The studies involving human participants were reviewed and approved by the Institutional Review Board of Wenzhou People’s Hospital. Written informed consent to participate in this study was provided by the participants’ legal guardian/next of kin.

## Author Contributions

All authors participated in the planning and execution of this study or analysis of the study data. TJ designed this study. All authors collected the data and conducted the relevant experiments. X-JC, LZ, E-DZ, and TJ collected the data and performed the statistical analyses. TJ and X-JC drafted the manuscript. LZ and E-DZ provided critical comments, suggestions and revised the manuscript. All authors contributed to the article and approved the submitted version.

## Funding

This study was supported in part by the Shanghai Sailing Program (No. 20YF1407500).

## Conflict of Interest

The authors declare that the research was conducted in the absence of any commercial or financial relationships that could be construed as a potential conflict of interest.
