# Supplementary material for: Pan-Cancer Analysis Identifies Liver Metastases as Negative Predictive Factor for Immune Checkpoint Inhibitors Treatment Outcome
Source: Front Immunol. 2021 Jun 24;12:651086. doi: 10.3389/fimmu.2021.651086 (PMC8264582; doi:10.3389/fimmu.2021.651086)
Supplement: Supplementary file 1 [file DataSheet_1.docx]

**Supplementary** **Text S1．**

**Methodology of meta-analysis**

**Publication online search**

We performed a comprehensive publication search through PubMed/Medline, EMBASE, Google Scholar, Cochrane Library and Web of Science until December 31, 2020 by using “lung cancer” and “PD-L1” and “liver” or their related words. Titles and abstracts were firstly reviewed to determine publications We then collected the data on the relationship of liver metastasis with overall survival (OS) and progression-free survival (PFS) in NSCLC patients treated with anti-PD-1/PD-L1 based treatments. Relevant conferences abstracts and presentations including the American Society of Clinical Oncology, the American Association for Cancer Research, the European Society for Medical Oncology and the World Lung Cancer Conference were also searched until December 31, 2020. We also manually screened the references of each eligible study until no additional articles could be added. This analysis was conducted in line with Preferred Reporting Items for Systematic Reviews and Meta-Analyses: the PRISMA Statement.

**Publication selection**

Publications met the following criteria were eligible: (1) randomized controlled trials (RCTs)that evaluated the efficacy of anti-PD-1/PD-L1 based treatments in patients with advanced NSCLC; (2) subgroup analysis included the liver metastatic group ; (3) reported data could analyze the presence of liver metastasis and/or high risk (HR) on clinical outcomes including PFS and OS. Studies were ineligible if they were: (1) retrospective studies, cost effectiveness analyses, quality of life studies, single-arm phase I or II trials, comment, reviews, case-only studies, editorial, or familial studies; (2) insufficient data for analysis of rate and/or HR with 95% confidence intervals (CIs); and (3) repeat of previous publications or replicated samples. When duplicate publications for the same study occurred, we included only the most recent and complete publications or the ones supporting the approval by the US Food and Drug Administration (FDA). The study eligibility was independently evaluated by two reviewers. Disagreements were resolved by discussion.

**Data extraction**

Three authors (T.J., X.J.C. and L.Z.) independently carried out the data extraction based on PRISMA statement by using a standardized data collection form. We extracted the following information from the included studies: trial name, published year, National Clinical Trials (NCT) identification number, trial phase, total patients, histological type, anti–PD-(L)1 drugs, lines of treatment, treatment group, primary endpoint, and follow-up duration. To avoid the selection bias, we did not utilize the data from the Kaplan-Meier curves. Two reviewers (Y.B and T.J.) independently extracted the data by using a predefined Excel form. They also independently extracted the hazard ratios (HRs) and the related 95% confidence intervals (CIs) for PFS and OS. Where available, we included the most updated survival data. Disagreements were solved by consensus.

**Quality assessment**

The methodologic quality for each included study was assessed according to the Cochrane Collaboration handbook based on the original publication or its update and the supplemental materials. The adequacy of the following aspects was evaluated: random sequence generation, allocation concealment, blinding of participants, blinding of outcome assessment, incomplete outcome data, selective outcome reporting, and other potential threats to validity. The risk of bias of each aspect was classified as low, high, or unknown. Discrepancies in data extraction and quality assessment were resolved by discussion to achieve consensus among all investigators.

**Statistical analysis**

95% CIs were determined per estimate and presented in forest plots. For time-to-event data, the HRs with related 95% CIs were directly extracted from the eligible publications or calculated using previous methods proposed by Tierney et al. Cochran's Q test and I^2^ statistic were used to determine the heterogeneity of different studies. Low-level heterogeneity was defined as *P* > 0.1 for the χ2 test and I^2^ < 25%. If the heterogeneity was non-significant, a pooled effect was calculated with a fixed-effects model. A random-effects model was used when the heterogeneity was statistically significant. Publication bias was assessed by using funnel plots, Begg’s and Egger’s tests. Statistical analysis was conducted by Review Manager 5.0 software and STATA v12.0 (Stata Corporation, TX). *P* values were two-sided and considered significant if < 0.05 except for the Q-test.

**Supplementary** **Table S1．Search Strategies**

Search included: PubMed and EMBASE: date was from the inception through June 2017

**1) PubMed search strategy**

| 1. "Lung neoplasms"[Mesh] |
| --- |
| 1. Lung cancer [Title/Abstract] |
| 1. Lung tumor [Title/Abstract] |
| 1. Lung tumour [Title/Abstract] |
| 1. Lung carcinoma* [Title/Abstract] |
| 1. Lung neoplas* [Title/Abstract] |
| 1. Lung malignan*[Title/Abstract] |
| 1. "B7-H1 antigen" [Mesh] |
| 1. "CD274 protein" [Mesh] |
| 1. PD-L1[Title/Abstract] |
| 1. PDL1[Title/Abstract] |
| 1. B7-H1 [Title/Abstract] |
| 1. CD274[Title/Abstract] |
| 1. "liver metastasis"[Mesh] |
| 1. "hepatic metastasis"[Mesh] |
| 1. liver metastasis [Title/Abstract] |
| 1. hepatic metastasis [Title/Abstract] |
| 1. (1 OR 2 OR 3 OR 4 OR 5 OR 6 OR 7) AND (8 OR 9 OR 10 OR 11 OR 12 OR 13) AND (14 OR 15 OR 16 OR 17) |

**2) EMBASE search strategy**

| 1. 'lung neoplasm'/exp |
| --- |
| 1. lung cancer:ab,ti |
| 1. lung tumor:ab,ti |
| 1. lung tumour:ab,ti |
| 1. lung carcinoma:ab,ti |
| 1. lung neoplas*:ab,ti |
| 1. lung malignan*:ab,ti |
| 1. lung adenoma*:ab,ti |
| 1. 'B7-H1 antigen'/exp |
| 1. B7-H1 |
| 1. 'CD274 protein'/exp |
| 1. CD274 |
| 1. 'PD-L1'/exp |
| 1. PDL1:ab,ti |
| 1. 'liver'/exp |
| 1. 'metastasis'/exp |
| 1. 'liver metastasis'/exp |
| 1. Liver metastasis:ab,ti |
| 1. Hepatic metastasis:ab,ti |
| 1. (1 OR 2 OR 3 OR 4 OR 5 OR 6 OR 7 OR 8) AND (((9 OR 10) OR (11 OR 12)) OR 13 OR 14) AND (15 OR 16 17 OR 18 OR 19) |

| **Table S1. Multivariate analyses of clinical parameters on PFS and OS in real-world cohort.** | | | | | | | |
| --- | --- | --- | --- | --- | --- | --- | --- |
|  | **PFS** | | |  | **OS** | | |
| **Factor** | **HR (log rank)** | **95% CI** | ***P* value** |  | **HR (log rank)** | **95% CI** | ***P* value** |
| Sex (Female/male) | 1.420 | 0.832-2.425 | 0.199 |  | 1.420 | 0.653-3.091 | 0.377 |
| Age (≥ 65/< 65) | 1.166 | 0.810-1.677 | 0.409 |  | 1.087 | 0.660-1.792 | 0.742 |
| Smoking (Smoking/Never) | 0.665 | 0.428-1.034 | 0.070 |  | 1.043 | 0.530-2.053 | 0.903 |
| PS (1-2/0) | 1.457 | 0.710-2.992 | 0.305 |  | 1.036 | 0.401-2.677 | 0.941 |
| Stage (IIIB/IV) | 0.661 | 0.336-1.299 | 0.229 |  | 0.685 | 0.307-1.527 | 0.355 |
| Histology (Non-adeno/Adeno) | 1.269 | 0.883-1.823 | 0.197 |  | 1.198 | 0.724-1.984 | 0.482 |
| Treatment line (>1/1) | 1.694 | 1.086-2.643 | 0.020 |  | 2.154 | 1.091-4.253 | 0.027 |
| Liver metastasis (yes/>no) | 1.546 | 1.037-2.551 | 0.039 |  | 1.543 | 1.011-2.936 | 0.046 |
| HR: hazard ratio; CI: confidence interval; Adeno: adenocarcinoma; PS: performance score. | | | | | | | |

| **Table S2. Baseline features of included studies.** | | | | | | | | | | |
| --- | --- | --- | --- | --- | --- | --- | --- | --- | --- | --- |
| **Trial ID** | **Year** | **NCT Number** | **Phase** | **No. patients** | **Histological type** | **PD-1/PD-L1 antibody** | **Treatment line** | **Treatment group** | **Primary endpoint** | **Follow-up (mo)** |
| CheckMate 017 | 2015 | NCT01642004 | III | 272 | Squamous NSCLC | Nivolumab | Second | Monotherapy | OS | 40.3 |
| CheckMate 057 | 2015 | NCT01673867 | III | 582 | Nonsquamous NSCLC | Nivolumab | Second or above | Monotherapy | OS | 40.3 |
| IMpower130 | 2019 | NCT02367781 | III | 723 | Nonsquamous NSCLC | Atezolizumab | First-line | Atezolizumab plus chemotherapy | OS and PFS | 18.5 |
| IMpower131 | 2019 | NCT02367794 | III | 683 | Squamous NSCLC | Atezolizumab | First-line | Atezolizumab plus chemotherapy | OS | 25.5 |
| IMpower132 | 2019 | NCT02657434 | III | 578 | Nonsquamous NSCLC | Atezolizumab | First-line | Atezolizumab plus chemotherapy | OS and PFS | 14.8 |
| IMpower133 | 2018 | NCT02763579 | III | 403 | SCLC | Atezolizumab | First-line | Atezolizumab plus chemotherapy | PFS and OS | 13.9 |
| IMpower150 | 2018 | NCT02366143 | III | 800 | Nonsquamous NSCLC | Atezolizumab | First-line | Atezolizumab plus chemotherapy plus bevacizumab | OS and PFS | 15.4 |
| KEYNOTE-189 | 2018 | NCT02578680 | III | 616 | Nonsquamous NSCLC | Pembrolizumab | First-line | Pembrolizumab plus chemotherapy | OS and PFS | 23.1 |
| KEYNOTE-604 | 2020 | NCT03066778 | III | 453 | SCLC | Pembrolizumab | First-line | Pembrolizumab plus chemotherapy | PFS and OS | NA |
| OAK | 2017 | NCT02008227 | III | 850 | NSCLC | Atezolizumab | Second or above | Monotherapy | OS | 21.0 |
| POPLAR | 2016 | NCT01903993 | II | 287 | NSCLC | Atezolizumab | Second or above | Monotherapy | OS | 14.8 |
| PFS, progression-free survival; OS, overall survival; NA, not applicable. | | | | | | | | | | |

| **Supplemental Table S3. The methodologic quality assessment for each included study.** | | | | | | | |
| --- | --- | --- | --- | --- | --- | --- | --- |
| **Trial ID** | **Random sequence generation** | **Allocation concealment** | **Blinding of participants** | **Blinding of outcome assessment** | **Incomplete outcome data** | **Selective outcome reporting** | **Other potential threats to validity** |
| CheckMate 017 | low | low | low | low | low | low | low |
| CheckMate 057 | low | low | low | low | low | low | low |
| IMpower130 | low | low | low | low | low | low | low |
| IMpower131 | low | low | low | low | low | low | low |
| IMpower132 | low | low | low | low | low | low | low |
| IMpower133 | low | low | low | low | low | low | low |
| IMpower150 | low | low | low | low | low | low | low |
| KEYNOTE-189 | low | low | low | low | low | low | low |
| KEYNOTE-604 | low | low | low | low | low | low | low |
| OAK | low | low | low | low | low | low | low |
| POPLAR | low | low | low | low | low | low | low |

**
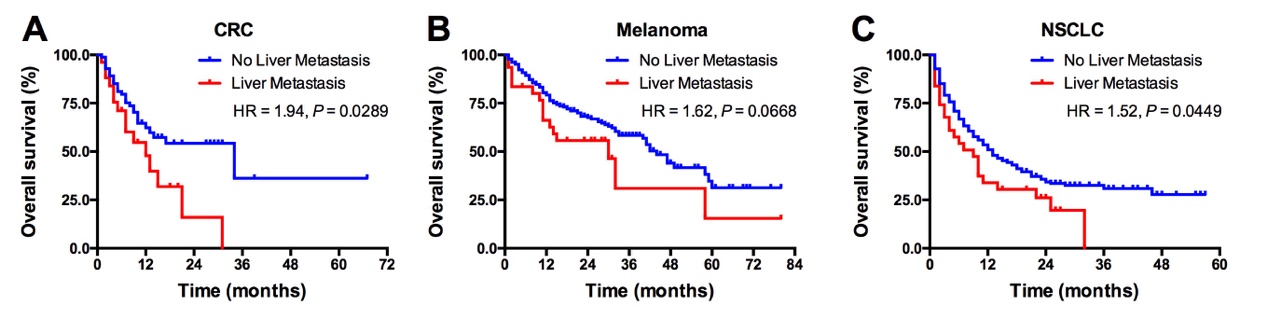
**

**Supplemental Figure S1. The predictive value of LM for ICIs treatment outcomes in colorectal cancer (A), melanoma (B) and non-small-cell lung cancer (C).**

**
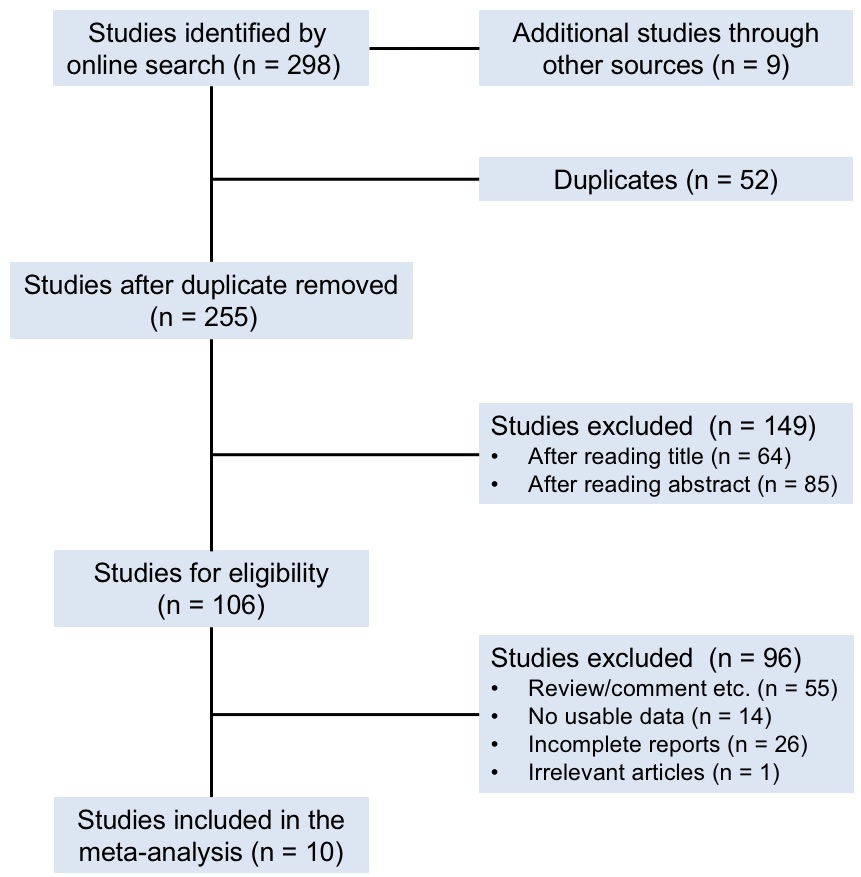
**

**Supplemental Figure S2. Flowchart of published studies’ identification.**

**
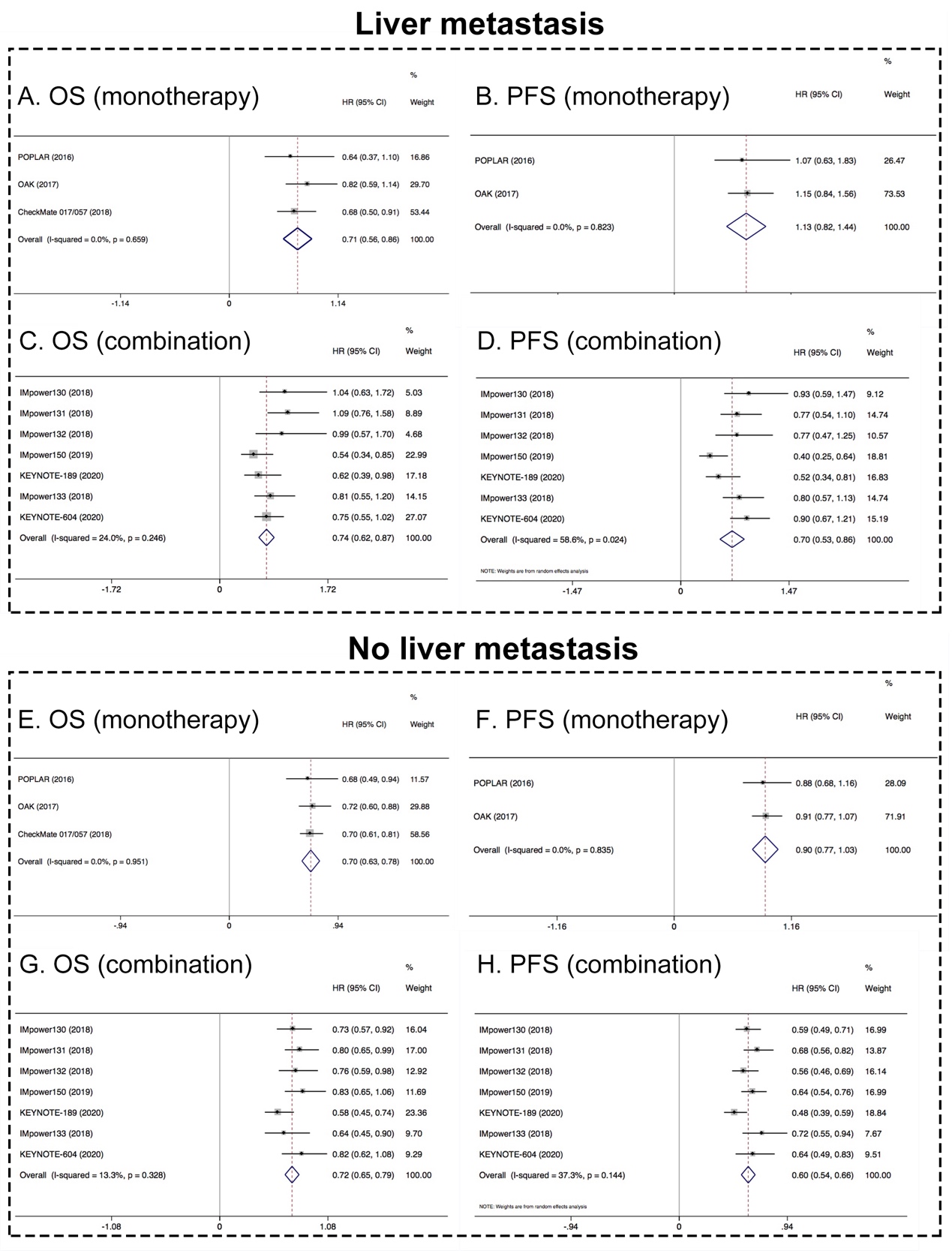
**

**Supplemental Figure S3. Subgroup analysis to evaluate the predictive value of LM in NSCLC treated with ICIs. A.** Pooled analysis of OS in patients with LM received ICI monotherapy; **B.** Pooled analysis of PFS in patients with LM received ICI monotherapy; **C.** Pooled analysis of OS in patients with LM received ICI based combination therapy; **D.** Pooled analysis of PFS in patients with LM received ICI based combination therapy; **E.** Pooled analysis of OS in patients without LM received ICI monotherapy; **F.** Pooled analysis of PFS in patients without LM received ICI monotherapy; **G.** Pooled analysis of OS in patients without LM received ICI based combination therapy; **H.** Pooled analysis of PFS in patients without LM received ICI based combination therapy. LM, liver metastasis.
